# Spirulina as a daily nutritional supplement of young pre-school Cambodian children of deprived settings: a single-blinded, placebo-controlled, cross-over trial

**DOI:** 10.1186/s12887-022-03766-5

**Published:** 2022-12-07

**Authors:** Hubert Barennes, Laetitia Houdart, Caroline de Courville, Florent Barennes

**Affiliations:** 1 Agence Nationale de Recherches sur le SIDA et les Hepatites Virales, Paris, France; 2 Antenna, 135 Street 95, Phnom Penh, Cambodia; 3 Institut Pasteur du Cambodge, 5 Preah Monivong Blvd, Phnom Penh, Cambodia

**Keywords:** Algae, anemia, Blood cell count, Cambodian, Children, Clinical trial, Growth, Health, Malnutrition, Nutrition, Prevention, Preschool, Spirulina, Supplements

## Abstract

**Background:**

Spirulina (SP) is widely used as a nutritional supplement to enhance child nutrition in low-income countries. We assessed Spirulina’s efficacy of the current dose supplied by institutions in Cambodia on improving growth and anemia in a cross-over randomized controlled trial in preschool underprivileged children from similar settings.

**Methods:**

Preschool children cared by a not-for-profit institution were randomly and blindly allocated (2 to 1) to spirulina or placebo: 100 g in total, given in 2 g per day. After 5 weeks of wash-out, participants were crossed-over to the other group. Anthropometric gain and selected hematological data (blood cell count, ferritin, and C-reactive protein) were assessed at each phase.

**Results:**

A total of 179 children completed the trial, 149 (83.2%) completed all the anthropometrics, and 99 (55.3%) all hematological measures. Mean BMI was 14.18 (95%CI: 14.00–14.37) and 31(20.8%) children had thinness. Mean blood hemoglobin was 11.9 g/dL (95%CI: 11.8–12.1). The weight gain of the SP group showed a modest higher trend compared to placebo (0.63 kg; 95%CI: 0.54–0.72 and 0.46 kg; 95%CI: 0.33–0.58, respectively; *p* = 0.07). Height increased similarly in both groups. The number of anemic children decreased by 6 (6.06%) and 11 (11.11%) on Placebo or SP, respectively (*p* = 0.004). Tolerance was good.

**Conclusion:**

SP may be recommended to improve childhood anemia. The analysis of the usual daily dose (2 g) provided by organizations in Cambodia shows a tendency to improve weight gain in the group supplemented with SP very close to significance, but no trend in height. Increased doses and longer supplementation should be evaluated further.

**Trial registration:**

The study was retrospectively registered at ISRCTN under number 11696165 on 12/12/2018.

**Supplementary Information:**

The online version contains supplementary material available at 10.1186/s12887-022-03766-5.

## What is already known on this topic?

Spirulina (SP) is a rich nutrient widely used as a nutritional supplement. There is some evidence for the effectiveness of SP (1 g per day) in alleviating vitamin A deficiency, and some debated evidence for improving the health of malnourished children.

Controlled studies of MS in children are very rare and the effectiveness of low daily doses has not been evaluated in improving growth in disadvantaged children.

## What this study adds?

Daily small doses of SP alleviated anemia.

The usual daily dose (2 g) provided by institutions in Cambodia shows a tendency to improve weight gain in the SP supplemented group without obtaining any significance (*p* = 0.07).

The results suggest to further evaluate doses and longer supplementation together with more subtle biological parameters of nutrition and immune function in a similar context of underprivileged children.

## Introduction

Spirulina (*Arthrospira platensis*) (SP), a filamentous microalga (Cyanobacterium) grows naturally in alkaline water bodies in subtropical waters [1]. Its extreme popularity [2, 3] as nutritional supplement is due to its improved bioavailability [4] and high contents content of balanced proteins (55–70%) polyunsaturated fatty acids, microelements, β-carotene, and other vitamins [5–14].

SP has been considered “the best food for the future”, to combat malnutrition in low-income countries and to improve overall health [13–16]. This resulted in extended scientific interest [5, 17] and its use by many institutions in developing countries [18–25]. SP is recommended for use as an ingredient in foods, at levels ranging from 0. 5 to 3. 0 g per serving [14, 25–27].

The rationale for using SP to correct anemia is based on SP’s high content of iron with high bioavailability, content of porphyrin, and its content of phycocyanin which boost the erythropoietic response [28–31]. Animal testing and clinical studies, mostly uncontrolled [32, 33], suggest a beneficial effect of SP [27–33]. However, control studies remain scarce.

In Cambodia, SP is provided to children by NGOs under the assumption that using low doses of SP daily (2 g) will improve the children’s growth and health. We assessed the impact of SP supplementation on growth, and anemia using a cross-over design in a single-blinded trial (Trial ID: ISRCTN11696165) and attempted to present this study according to the consort check-list (S1. Cohort check-list).

## Methods

### Survey participants

The survey was conducted at the preschool “Pour un Sourire d’Enfant (PSE)” in Phnom Penh, Cambodia from October 2014 until July 2015. Children (4–7 years) were included if they had no current disease, or allergy to SP, were able to attend the PSE school, and with the informed oral consent of their parents/guardians after a medical screening at PSE health center. All PSE children are fed once daily at school.

### Randomization and blinding

A randomized list using computer generated numbers by blocks of four was prepared before the trial and secured in an external office outside PSE. Envelops with the study number and group name inside were prepared by an external assistant, a pharmacist trainee who was not involved in the distribution of the envelopes, and then sealed. A number corresponding to the identification code and class number of the child was recorded on the envelope following the randomization list. As a result, no connection between these codes and the name of the children was possible for the people preparing the envelopes. Individual envelopes were given to each class according to the number of participants and opened by the teachers under the supervision of the team. SP and placebo were provided as sprinkles which had no attractive taste. The placebo was brown unsweetened dietary sprinkles available locally. Due to this small minimal difference, the study was downgraded from double to single blind trial. Everything was done to be close to a double-blind clinical trial. The authors had no information on the individual status of children on SP or placebo before and during the study. In addition, blinding was ensured for the trial team during data entering and preliminary analysis using a code number 0 or 1 based on the nutrient allocation.

SP was obtained from Antenna Cambodia, a non-for- profit organization highly active in SP field which conducts regular quality control [23–25, 28]. Assessment for potential bacterial contamination content was conducted by Pasteur Institute, Phnom Penh, and for iron and vitamin A content by Laboratoire Developpement Méditerranée AQMC lab Saint-Aunes France. Both analyses were satisfactory. Iron and vitamin A contents were at 666 mg/100 g and 1 μg/100 g, respectively.

### Baseline assessments

Baseline anthropometric measures and hematological assessments were conducted before the survey, then 4 weeks after the first treatment the week just before Phase 2, and after the second supplementation. Anthropometric assessment (weight and height, using UNICEF tools with 100 g and 0. 1 precision, respectively) was conducted by the same person after rigorous training and testing. Weight for height (Wasting) score and Height for age score (undernutrition) were calculated using WHO Anthro software for children below 5 years. Body mass index (BMI) for children using WHO 2007 standards for children below and above 5 years was calculated.

Laboratory blood samples (4 ml) were taken by nurses from the Cambodian Pasteur Institute. The samples were stored and assayed for blood cell count, hemoglobin level (HB, mean corpuscular volume (MCV), and mean corpuscular hemoglobin (MCH) using Pentra 80XL (HORIBA), ferritin using Elecsys E411(ROCHE), and C-reactive protein (C-RP) using Pentra C400 (HORIBA) at Pasteur Institute laboratory.

### Supplementation and follow up

Two grams of SP or Placebo was administered daily by the children’s teacher during the first hour of the morning 5 times a week for 8 weeks or until the end of the individual bag of 100 g.

After 5 weeks of the wash-out period, participants were crossed over to the other group (Phase 2). The intake and tolerance of supplements were directly observed (DOT) by the teachers and events were recorded daily on a standardized pre-tested one-sheet form during Phase 1. This form recorded events such as nausea, vomiting, or any signs of potential allergy or side effects. The form was revised daily during the first weeks of the trial. Children with non-minor events had to be presented directly to the school health center.

### Outcome

The main outcome was the change in nutritional status, specifically a difference in weight and height before and at the end of the trial. Secondary outcomes were the tolerance and the evolution of hematological data.

### Definitions

Anemia was defined as hemoglobin below 11 g/l using WHO categories[46]. Eosinophil levels were defined as increased > 500 per mm3. C-RP was defined as normal if < 6 mg / l. Ferritin was defined as low if < 30 μg / L. Malnutrition was appreciated using weight for height and height for age z-score in children below 5 years.

### Study size

A required sample size of 119 people was calculated to detect a treatment difference of 0. 3 with alpha risk = 0. 05 and power 90%, based on the assumption that the standard deviation of the difference in the response variables is 1 with A ratio of 2:1 (SP)/(Control).

One of the constraints of the trial was the anticipated dropping out of preschool children. Finally, it was decided to include 50% more children in case of drop-out or incomplete data.

### Analysis

Data was entered using Epidata 7 and analyses were conducted using Stata11. Analysis was per protocol (PP) for the main outcome and anemia and by intention to treat (ITT) for the secondary outcome of tolerance. For comparison of means, an analysis of equality of variance and an analysis of normality of variables (Shapiro-Wilk W test) were conducted. Students’ t-test for unpaired variables or the Wilcoxson test were used as appropriate. Fisher’s exact test and Pearson chi-square were used to comparing frequency. The pairwise comparisons between Placebo and SP group were done using paired Students’ t-test and Wilcoxon signed-rank test, and the McNemar test for frequency. *P* < 0. 05 was deemed as statistically significant.

### Patient and public involvement

Patients or the public were not involved in the design, or conduct, or reporting, or dissemination plans of this research.

## Results

### Initial characteristics of children

**Fig. 1:**
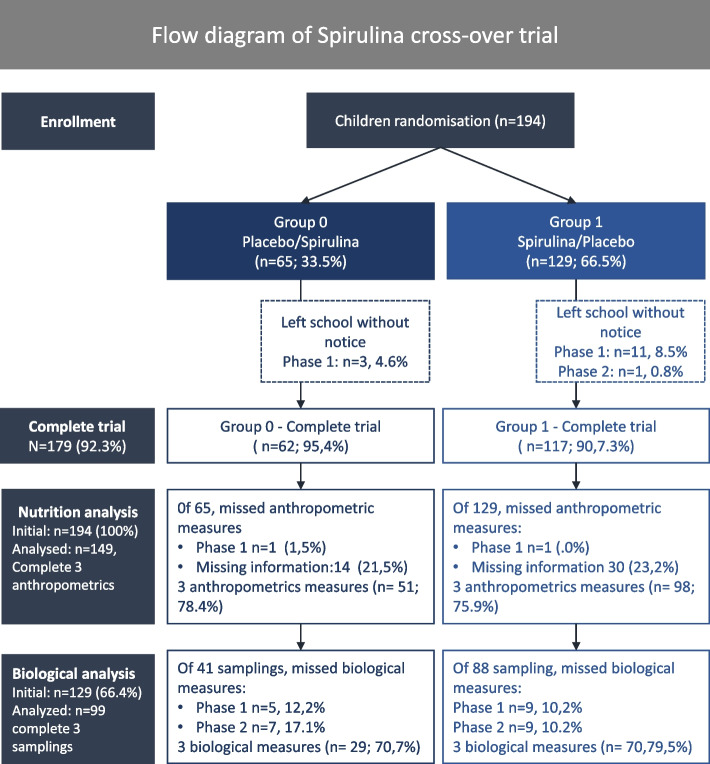
Flow chart of children included in the placebo-controlled spirulina trial

A total of 194 children were included in the survey (Fig. 1). No refusal from parents was recorded. A total of 15 children withdrawn from the survey, 14 during Phase 1 and 1 during Phase 2, leaving 179 (92. 3%) for safety analysis. Of them, 149 (83. 2%) completed the three anthropometrics satisfactorily and were analyzed. Of 129 children present for initial blood sampling, 99 underwent the three laboratory measurements and were analyzed.

No significant difference was encountered at baseline between the initial randomized groups 0 and 1 of children who completed the study (Table 1). The initial mean weight and height were 14. 2 kg (95%CI: 13. 9–14. 6) and 100. 2 cm (95%CI: 99. 0–101. 3), respectively. The mean BMI was 14. 18 (95%CI: 14. 00–14. 37) and 31 (20. 8%) children had thinness. Of the 102 children aged below 5 years, 16 (15. 7%) were suffering from wasting and 23 (22. 5%) from stunting. The mean blood hemoglobin was 11. 9 g/dL (95%CI: 11. 8–12. 1), 11 children had light anemia (11. 11%), 32 (32. 32%) microcytosis and 82 (82. 8%) children had hypereosinophilia, 9 (9. 1%) has a low ferritin and C-Reactive Protein (CRP) above 6 mg/L. The initial characteristics of enrolled children are presented (Supplementary Tables 1 and 2).

**Table 1:** Baseline parameters of participants enrolled in the spirulina placebo-controlled trial

Nutrition (***n*** = 149)	Group 0	95% CI	Group 1	95% CI	*p*
	*n* = 51		*n* = 98		
Order	Control/SP		SP/control		
Female	26	50.9%	44	44.9%	0.5
Age	4.3	4.0–4.6	4.3	4.1–4.5	0.9
Weight (kg)	14.0	13.4–14.6	14.4	14.0–14.8	0.3
Height (cm)	99.6	97.5–101.6	100.5	99.1–101.9	0.4
BMI	14.1	13.7–14.3	14.2	13.9–14.4	0.4
Thinness BMI < − 2 SD	12	23.5%	19	19.4	0.3
Child under 5 years (*n* = 102)	35		67		
Wasting: Weight/height < −2	9	25.7%	7	10.4%	0.07
Weight/height < −1	9	25.7%	29	43.2%	
Stunting: Height/Age < −2	10	29.4%	13	19.4%	0.5
Height/Age < −1	8	23.5%	18	26.8%	
**Hematology (*n*** **= 99)**	*n* = 29		*N* = 70		
Hemoglobin (g/dL)	12.1	11.8–12.4	11.8	11.7–12.0	0.2
Child with anemia^a^*	2	6.9%	9	12.8%	0.3
Eosinophil (/mm^3^)	1578	1156–2001	1445	1129–1761	0.2
Ferritin (μg/L)	71.93	47.98–72.70	67.74	59.8–75.3	0.9
Ferritin ≤30 μg/L^b^	2	6.8%	7	10%%	0.6
C-RP1(mg/L) *	1.8 [1]	0.8–2.7	1.5[4]	1.0–2.1	0.2
C-RP ≥ 6 mg/L *	2	(6.9%)	7	10.0%	0.6

Mean and 95% confidence interval. Frequency and (%). BMI: Body mass index. Thinness was defined according to the BMI WHO 2007 tables for children. ^**a**^if hemoglobin ≤11 g/dL. ^b^defining ferritin deficiency. * *p*-Value based on Wilcoxon signed-rank test or MacNemar’s Chi2 on within-subject differences between Spirulina and Placebo. C-RP: C-reactive protein

### Changes during the administration of placebo and Spirulina

The absolute changes within the Placebo and Spirulina groups are shown in Table 2. The weight gain of the SP group showed a modest benefit compared to Placebo, which was close to the significance level (0. 63 kg; 95%CI: 0. 54–0. 72 and 0. 46 kg; 95%CI: 0. 33–0. 58, respectively; *p* = 0. 07). Height increased similarly in both groups (2. 6 cm 95%CI: 1. 7–3. 4 and 2. cm 95%CI: 1. 9–2. 2, respectively; *p* = 0. 8). Thinness was appreciated for the whole population while wasting and stunting were appreciated among the 60 children aged below 5 years at the end of the study. The proportion of children with thinness decreased more in the SP group without being significant (− 15% versus − 2. 14%, respectively; *p* = 0. 17). The proportion, of children aged below 5 years affected with wasting and stunting did not change between placebo and SP groups.

**Table 2:** Nutrition and hematological changes during administration of Placebo and Spirulina in the spirulina cross- over trial

	Placebo				Spirulina				*p*
	Baseline		Change		Baseline		Change		
Anthropometrics	(n = 149)	95%CI	(n = 149)	95%CI	(n = 149)	95%CI	(n = 149)	95%CI	
Weight (kg) ^μ^	14.80	14.42–15.18	0.50	0.39–0.62	14.55	14.18–14.92	0.62	0.53–0.72	**0.07**
Height (cm) ^μ^*	101.67	100.50–102.83	2.61	1.76–3.46	100.90	99.80–102.13	2.12	1.99–2.26	0.8
Thinness: BMI -2SD^μ^ *	42	28.19%	−3	−2.14%	55	36.91	− 21	−15%	0.17
Child under 5 years at end (*n* = 60)									
Wasting: Weight/height < − 2 ^a^ *	10	16.6%	2	3.3%	9	15.00%	0		0.3
Weight/height < −1	21	20.58%	−5	8.33	21	35.00%	5	8.33	
Stunting: Height/Age < −2	11	18.3%	1	1.6%	10		0		0.6
Height/Age < −1	10	16.6%	3	5%	12		1		
Biology	(n = 99)	95%CI	(n = 99)	95%CI	(n = 99)	95%CI	(n = 99)	95%CI	
Hemoglobin (g/dL)*	11.87	11.68–12.05	.09	−0.04; 0.23	11.86	11.69–12.02	.01	−0.11; 0.13	0.4
Children with Anemia (< 11 g/d)*	15	(15.15%)	−6	(−6.06%)	12	(12.12%)	−11	(−11.11%)	**0.004**
Eosinophil (/mm^3^)*	1209	988–1430	− 287	− 424; − 151.1467	1319	1074–1565	− 282	− 437; − 128	0.9
Children with hyper eosinophil*	73	73.74%	− 9	− 9.10%	80	80.81%	− 11	− 12.8%	0.4
Ferritin (μg/L)*	62.40	54.96–69.84	3.10	−9.21; 15.42	65.57	58.87–72.27	−2.65	−8.87; 3.56	**0.06**
Ferritin < 30 μg/L*	14	14.14%	4	4.04%	10	10.10	3	3.03%	1
MCV (fL)	73.69	72.46–74.92	−.059	−0.39-0.27	73.63	72.35–74.91	−.04	− 0.36 - 0.26	0.9
MCV ≤ 70	26	26.26%	0	–	22	22.22%	+ 5	5.15%	1
C-Reactive Protein (mg/L)*	1.29	0.90–1.67	0.34	−0.28; 0.96	1.84	1.25–2.43	−.001	−.73; 0.73	
Children with CRP > 6	4	(4.04%)	4	(4.04%)	8	(8.08%)	−1	(− 7.07%)	0.09

Statistically significant results or results of interest are highlighted in bold text. Values are mean and 95%CI or frequency and (%) as appropriate. Anemia (≤11 g/d); Hyper eosinophilia ≥500 Eosinophil (/mm^3^). ^μ^ < 0.05 between groups at baseline due to the ratio of the group number (1 to 2). * *p*-Value based on Wilcoxon signed-rank test or MacNemar’s Chi2 on within-subject differences between Spirulina and Placebo

^a^60 children were aged below 5 years at the end of the study and could be analyzed with WHO weight for height and height for age Z-scores

Few children had anemia in both groups (15, 15. 1% and 12, 12. 1% in Placebo and SP groups, respectively). There was a significant decrease of the number of children with anemia in the SP group compared to Placebo group. The number of anemic children decreased by 6 (6. 0%) and 11 (11. 1%) in the placebo and spirulina groups, respectively (*p* = 0. 004).

No significant change was observed in the change of mean hemoglobin, mean MCV, mean eosinophils or, in the number of children with low ferritin or microcytosis between Placebo and SP. The number of children with hyper eosinophils decreased slightly similarly since the beginning of the trial and remains high despite a deworming treatment given to all children by the medical health Centre before Phase 2.

Two changes were close to the significance level of 0. 05. The mean ferritin increased in placebo group while it decreased in SP group (*p* = 0. 06). The number of children with elevated C-RP which was low at the beginning of the trial, decreased in the SP groups from 8 to 1 and increased from 4 to 8 in the Placebo group (*p* = 0. 09).

### Acceptability and tolerance of supplements

The acceptability of both supplements was good. No refusal was reported by the teachers. Tolerance was good. Only a few transient and benign side effects of transient nausea or some vomiting were reported by the teachers during the first days in less than ten children with no detectable differences between the groups.

## Discussion

This survey provides the findings of a cross-over blind randomized control trial of 2 g of SP daily compared to a placebo given in underprivileged children cared for by PSE preschools in the Phnom Penh area of Cambodia. The trial was set up to explore the potential of SP as a booster to improve growth and hematological profile including anemia status. It shows a modest gain of weight, close to the significance level and a significant decrease in the number of children with anemia in the SP group compared to the placebo group. Two other outcomes changed and were close to significance level: the mean ferritin change and the number of children with elevated C-RP.

Frequent shortcomings of SP studies in children have been previously described, particularly the small size or the absence of a control group in most studies [16–18, 25]. We attempted to compensate for these shortcomings by using a cross-over design.

The gain in weight in the SP group compared to the Placebo group was modest. The short duration and minimal input of calories and proteins by 2 g of SP can be suspected to be the cause of the modest specific impact of SP on weight gain as previously discussed [21, 26]. The fact that all children benefited from a daily meal provided by the school probably decreased the interest of supplementing low doses of SP nutrients. There are no comparable controlled studies of SP supplementation in young underprivileged children attending preschool. Most studies in children were conducted on children with known health problems or older children [20, 27, 34–36]. A study in India among older schoolgirls used 0.9 g daily of SP for 3 months and suggested nutritional benefits, but this was not a controlled trial [32]. A controlled study in young Chinese schoolgirls showed the effectiveness of 2 and 4 g of daily SP for 10 weeks to improve vitamin A status [37]. In the context of severe malnutrition, the objectives are quite different and daily dosages of SP ranged from 1 g daily for 1 year for vitamin A deficiency, 3 g daily in a controlled study in Gaza, and up to 10 g daily for severe malnutrition [17–22, 26, 34–41]. In addition to doses, another question is the duration of SP supplementation. In Burkina Faso, a study compared a group of children receiving nutritionally enhanced flour and deworming treatment with two groups of children receiving an additional 5 g of SP for 90 days [21]. This study led to an unsatisfactory result of SP supplementation according to the author. The conclusions were highly criticized by various organizations [38–40]. Looking back at the results, we found that some of the interesting findings of this study and their implications might have been missed in the passionate debate. At 90 days, a weight gain persisted in the SP group while decreasing in the SP-free group. The persistence of weight gain at 90 days suggests that prolongation of supplementation could be beneficial for children when weight gain is the main objective. From these studies and our findings, it may be suggested that higher doses of SP can be considered in future studies in children from deprived settings beginning with 3 g daily of SP.

A significant decrease in the number of children with anemia was observed while on SP compared to the placebo group. The study shows a clear benefit of SP on the reduction of anemic compared to placebo and can be recommended. This result is supported by animal studies [28–31, 42, 43] and clinical studies [32, 33, 43, 44].

Further studies should consider assessing higher doses and longer supplementation periods together with more subtle biological parameters for nutrition and immunity, which we were unable to address due to limited funds.

### Limitations of the study

This study had to face an expected high rate of loss to follow up on this group of underprivileged children. We tried to compensate for this using a larger number of children than calculated.

Nutritional intake at home was unknown. This was mitigated by the fact that all children received their lunch at PSE. No stool testing was conducted, and the high level of parasitism reflected by the rate of hyper eosinophil persisted during the study despite a general deworming after the first phase. This was probably affecting the normal absorption of iron in children.

## Conclusion

The tolerance and acceptability of SP were good. SP 2 g daily may be recommended to improve childhood anemia. The usual dose of 2 g provided by institutions in Cambodia has only a modest impact on weight and no trends in height. Increased doses and longer supplementation should be evaluated to improve the growth of children, together with more subtle biological parameters of nutrition and immune function in a similar context of underprivileged children.

## Supplementary Information

**Additional file 1.**

## Data Availability

The datasets used and/or analysed during the current study are available from the corresponding author on reasonable request.
