# Supplementary material for: Spirulina as a daily nutritional supplement of young pre-school Cambodian children of deprived settings: a single-blinded, placebo-controlled, cross-over trial
Source: BMC Pediatr. 2022 Dec 7;22:701. doi: 10.1186/s12887-022-03766-5 (PMC9727933; doi:10.1186/s12887-022-03766-5)
Supplement: Supplementary file 1 — Additional file 1. [file 12887_2022_3766_MOESM1_ESM.docx]

S1 Table: Initial characteristics of children included in the placebo-controlled spirulina study

|  | **Group 0**  **n=65** | **95 % CI** | **Group 1**  **n=129** | **95 % CI** | **p** | **Total**  **n=194** | **95 % CI*** |
| --- | --- | --- | --- | --- | --- | --- | --- |
| **Baseline** | **Control** |  | **spirulina** |  |  |  |  |
| Female* | 32 | 49.2% | 56 | 43.4 | 0.4 | 88 | 45.4 % |
| Age (years)** | 4.8 | 4.5- 5.2 | 4.9 | 4.6-5-1 | 0.9 | 4.8 | 4.6–5.0 |
| Weight1 (kg) | 14.4 | 13.8- 14.9 | 14.7 | 14.3-15.1 | 0.3 | 14.6 | 14.2-14.9 |
| Height1 (cm) | 100.7 | 98.7-102.6 | 101.3 | 100.0-102.5 | 0.6 | 101.1 | 100.0-102.1 |

* Frequency and %. ** age: available for 123 children. Mean and 95% confidence Interval.

S 2: Baseline biological characteristics of children sampled initially enrolled in the spirulina placebo-controlled study

|  | Group 0  N=41 | | Group 1  N=88 | |  | Total  n=129 | |
| --- | --- | --- | --- | --- | --- | --- | --- |
| Variable | Mean | 95% CI | Mean | 95% CI | p | Mean | 95% CI |
| Hemoglobin **(g/dL)** | 12.18 | 11.9-12.4 | 11.87 | 11.6-12.0 | 0.05 | 11.9 | 11.8-12.1 |
| Hb <11g **/dL*** | 2 | (4.8%) | 13 | (14.7%) | 0.1 | 15 | (11.6%) |
| Leukocytes | 10729 | 9855-11602 | 10481 | 9931-11032 | 0.6 | 10560 | 10099-11021 |
| Eosinophil %^&^ | 12.7 | 10.6-14.8 | 12.2 | 10.5-14.0 | 0.7 | 12.4 | 11.0-13.7 |
| MCV **(fL)** | 74.41 | 72.5-76.2 | 73.77 | 72.3-75.1 | 0.5 | 73.9 | 72.8-75.0 |
| MCV ≤ 70****** | 9 | (21.95%) | 31 | (34.2%) | 0.4 | 40 | (31.0) |
| Ferritin (**µg/L)***** | 70.22 | 57.4-82.9 | 66.52 | 59.6-73.4 | 0.5 | 67.6 | 61.5-73.7 |
| C-RP (mg/L) | 1.77 | 1.0-2.4 | 1.62 | 1.1-2.0 | 0.7 | 1.6 | 1.3-2.0 |
| C-RP above 6 | 1 | (2.4%) | 6 | (6.7%) | 0.4 | 7 | (5.4%) |

Mean and 95% confidence interval or frequency and (%). Leukocytes = white blood cell count. MCV: Mean cell volume. C-RP: C-reactive protein. ^&^ 103 had hyperoesinophilia > 500 /mm^3^. * Number of children with hemoglobin <11g **/dL** defining anemia, ** Defining a microcytic anemia when associated with hb<11g/dL; ***Ferritin normal range: **H=30-280µg/L; F=20-120µg/L,**
